# Robust monolithic polymer(resorcinol-formaldehyde) reinforced alumina aerogel composites with mutually interpenetrating networks

**DOI:** 10.1039/c9ra03227d

**Published:** 2019-07-25

**Authors:** Ya Zhong, Gaofeng Shao, Xiaodong Wu, Yong Kong, Xue Wang, Sheng Cui, Xiaodong Shen

**Affiliations:** College of Materials Science and Engineering, Nanjing Tech University Nanjing 210009 PR China yzhong@njtech.edu.cn; Suqian Advanced Materials Institute of Nanjing Tech University Suqian 223800 PR China; Jiangsu Collaborative Innovation Center for Advanced Inorganic Function Composites Nanjing 210009 PR China; Chair of Advanced Ceramic Materials, Technische Universität Berlin Berlin 10623 Germany

## Abstract

Monolithic polymer(resorcinol-formaldehyde) reinforced alumina (RF/Al_2_O_3_) aerogel composites were prepared using a sol–gel method and supercritical fluid CO_2_ drying. The formation mechanism, chemical compositions, pore structures, morphologies, thermal and mechanical performances of RF/Al_2_O_3_ aerogel composites with different RF/Al molar ratios were investigated. The results show that the two networks of organic resorcinol-formaldehyde and inorganic alumina are completely independent of one another. The as-synthesized RF/Al_2_O_3_ aerogels consist of spherical organic carbon particles and fibrous alumina, which possess low bulk density (0.077–0.112 g cm^−3^), low shrinkage (1.55–2.76%), low thermal conductivity (0.024–0.028 W m^−1^ K^−1^), and high specific surface area (453.26–722.75 m^2^ g^−1^). Especially, the sample prepared with molar ratio RF/Al = 1 shows the best network structure with the higher compressive strength (1.83 MPa) and Young's modulus (122.57 MPa). The resulting robust RF/Al_2_O_3_ aerogel composites could be potentially used as thermal insulators, catalysts and adsorbents.

## Abstract 2

Monolithic polymer(resorcinol-formaldehyde) reinforced alumina (RF/Al_2_O_3_) aerogel composites were prepared using a sol–gel method and supercritical fluid CO_2_ drying.

## Graphical abstract

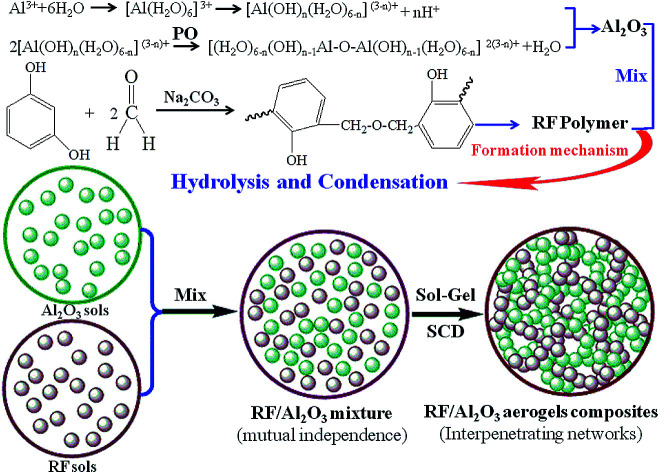

## 1. Introduction

Aerogels, a kind of magical nanomaterial, have been investigated for a wide variety of applications, including thermal insulation,^1^ catalysis,^2^ catalyst carrier,^3^ adsorbents,^4^ sensors^5^ and drug delivery systems,^6^ to name a few, because of their exceptional physical properties, such as extremely low-density (3–150 kg m^−3^), high porosity (85–99%) and high specific surface area (700–1300 m^2^ g^−1^).^7–9^ Generally speaking, silica and alumina aerogels exhibited stable nano-porous network structures, and are attractive candidates for thermal insulation.^10^ However, owing to their brittle nature and crystallization-induced pulverization behavior, conventional oxide aerogels often suffer from serious strength degradation and structural collapse under large thermal gradients or extended high-temperature exposure.^11^ Compared with silica aerogels, alumina aerogels possess better mechanical and chemical stability, endowing them with great potential applications.^12^ Unfortunately, it is still difficult for the mechanical properties of pure alumina aerogels to meet the requirements of practical applications as a result of the inherent three-dimensional network, which consists of alumina nanoparticles with diameters of 5–10 nm connected by narrow inter-particle necks.^13,14^ Therefore, robust mechanical and thermal resistance are the key roadblocks to using aerogel materials.

To address this issue, various structural reinforcement strategies have been attempted to improve the mechanical properties of aerogels. Incorporation of inorganic fibers,^15–22^ carbon fiber^23–25^ or advanced nanomaterials^26–31^ as supporting skeletons into aerogel matrixes is one of the most convenient and effective methods to overcome their fragility and poor mechanical properties.^32,33^ Moreover, appropriate fibers could not only strengthen the aerogel materials but also be used as opacifiers to reduce the radiative heat transport in aerogels at high temperature.^34^ Nevertheless, due to the relatively thicker diameter (5–25 μm) and the brittleness of inlaid inorganic fibers, most of the aerogel matrix usually crack into small fragments with impairing the integrality,^35^ destroying the microscopic pore structures and decreasing the mechanical properties of the aerogel composites.^36^

Hybridization of oxide aerogels with polymers is another extensive researched approach to obtain robust aerogels by increasing their tensile strength. Depending on the chemical relationships between the polymers and the surrounding skeletal structures, polymer/sol–gel composites are divided into two categories:^37^ (1) the polymer and the inorganic framework are completely independent of one another, namely, interpenetrating networks; (2) there is covalent bonding between the polymeric and the inorganic component, namely, crosslinking frameworks. Up to now, various polymers had been successfully integrated with oxide aerogels as reinforcement to improve their mechanical properties. For instance, Leventis *et al.*^38^ proposed a method using poly(hexamethylene diisocyanate) as cross-linker to prepare the strong lightweight silica/Di-ISO aerogel monoliths, which are much less hygroscopic than native silica and do not collapse when in contact with liquids. Moghaddas *et al.*^39^ developed a method of preparing the silica aerogel/rigid polyurethane foam nanocomposite by ambient pressure drying, which showed efficient thermal insulation (0.0268–0.0314 W m^−1^ K^−1^) and good mechanical properties. Hu *et al.*^40^ introduced a method of using poly(dimethylsiloxane) as reinforcement to prepare compressible and superhydrophobic polymer/graphene aerogel composites, which showed enhanced compressive strength and a stable Young's modulus. Özbakır *et al.*^41^ synthesized the novel monolithic and crack-free PMVE-silica aerogel composites by CO_2_ supercritical drying and the effect of polymer fraction in solid network on drying was investigated both by experiments and simulations. Li *et al.*^42^ fabricated silica aerogel/aramid pulp composites *via* ambient pressure drying by adding aramid pulps into silica sol directly, which retained the integrality and nice interface adhesion. The compressive strength was enhanced obviously up to 1.2 MPa and the low thermal conductivity of 0.0232–0.0278 W m^−1^ K^−1^. Maleki *et al.*^43^ introduced a low-cost and time-saving method of using BTMSH and ETESB as cross-linkers to prepare lightweight polymer-reinforced silica aerogels, which showed good compression strength (11–400 kPa) and low thermal conductivity (0.039–0.093 W m^−1^ K^−1^). Therefore, the polymer-reinforced aerogel composites exhibit excellent structural integrity and mechanical performance without sacrificing other unique properties. However, the research about polymer reinforced alumina aerogel is limited.

It is generally known that interpenetrating inorganic sol–gel networks with polymers have been pursued mainly for preventing the shrinkage and cracking problems encountered upon drying of the wet inorganic gels. In this study, monolithic polymer(resorcinol-formaldehyde) reinforced alumina (RF/Al_2_O_3_) aerogel composites were prepared using sol–gel method and supercritical fluid CO_2_ drying. In addition, the two networks of organic resorcinol-formaldehyde and inorganic alumina were completely independent of one another. Furthermore, the details of synthesis and discussion of the effects of RF/Al molar ratios on the microstructures evolution and physicochemical properties of RF/Al_2_O_3_ aerogel composites are given below.

## 2. Experimental

### 2.1 Chemicals

Resorcinol (R), formaldehyde (F, 37%w/w aqueous solution), aluminum chloride hexahydrate (Al), deionized water (H_2_O), absolute ethyl alcohol (EtOH), sodium carbonate (C) and propylene oxide (PO) were used as raw materials. All of the reagents and solvents are analytical grade and used as received without further purification.

### 2.2 Synthesis of RF/Al_2_O_3_ aerogel composites

RF/Al_2_O_3_ hybrid sols were prepared according to the following steps. RF(molar ratio, R/F = 1/2) Al, H_2_O, EtOH were directly mixed in a pot with a molar ratio of (0.5, 0.67, 1.0, 1.5, 2.0): 1 : 48 : 16, sodium carbonate was used as catalyst (molar ratio, R/C = 200), stirring for about 60 min at 50 °C for complete hydrolysis and then cooled down to room temperature. Subsequently, desired amounts of PO (molar ratio, PO/Al = 10) was slowly dropped into the clear solution (propylene oxide was transferred by syringe through a septum, so as to reduce laboratory exposure and ensure safety). After that, the reaction mixture was further stirred for 30 min at room temperature, transferred to plastic molds, and the solutions were allowed to gel at room temperature within 3 h. In order to increase the strength, the wet gels were firstly aged at room temperature for 72 h. Afterwards, the wet gels were demolded, aged in an air oven at 65 °C for 72 h, and simultaneously washed with ethanol every 24 hours to exchange the water and reaction byproducts from the pores of the samples. After aging and solvent exchange, the color of monolithic wet gels tune to opaque-red from transparent-red, and the alcohol gels were dried in an autoclave (HELIX 1.1 system, Applied Separations, Inc., Allentown, PA) with supercritical fluid CO_2_ to form RF/Al_2_O_3_. Finally, the as-synthesized samples were denoted as S_1_, S_2_, S_3_, S_4_ and S_5_, the corresponding RF/Al molar ratios of (0.5, 0.67, 1.0, 1.5, 2.0) : 1.

### 2.3 Measurements and characterizations

The samples were prepared in cylinders (diameter 25 mm, height 25 mm) and the bulk density of the aerogels was determined by *ρ* = *m*/*v* where *ρ*, *m* and *v* are bulk density, mass and volume (obtained by *v* = π*D*^2^*h*/4 where *D* and *h* are diameter and height of the aerogels) respectively. Thermal gravimetric analysis (TGA) and was performed by NETZSCH STA449C thermogravimetric analyzer under a constant nitrogen flow of 30 ml min^−1^ at a heating rate of 10 °C min^−1^ to 1200 °C. A Fourier-transform infrared (FT-IR) spectrum was recorded on a Bruker-Equinox 55 spectrophotometer in KBr pellets with a scanning range of 4000–400 cm^−1^. X-ray diffraction (XRD) patterns were carried out using an ARL X′ TRA diffractometer (Rigaku) with Cu-Kα radiation (30 kV, 30 mA). The microstructure was surveyed by LEO-1530VP scanning electron microscopy (SEM) and JEOL JEM-2010 electron microscope (TEM), operating at the acceleration voltage of 10 kV and 200 kV, respectively. Pore structure properties were measured by Nitrogen adsorption/desorption porosimetry (Micromeritics ASAP2020 surface area). The specific surface area was calculated using Brunauer–Emmett–Teller (BET) and the pore-size distribution was derived from the desorption branch of isotherms by using the Barrett–Joyner–Halenda (BJH) model. The thermal conductivities were tested using a Hot Disk Thermal Constants Analyzer (TPS2500S, Sweden). The compressive strengths and Young's modulus of the monoliths aerogels were measured by using an INSTRON 3382 testing machine. The test temperature was 25 °C and the test speed was 2.0 mm min^−1^.

## 3. Results and discussion

### 3.1 Formation mechanism of RF/Al_2_O_3_ aerogel composites

**Fig. 1:**
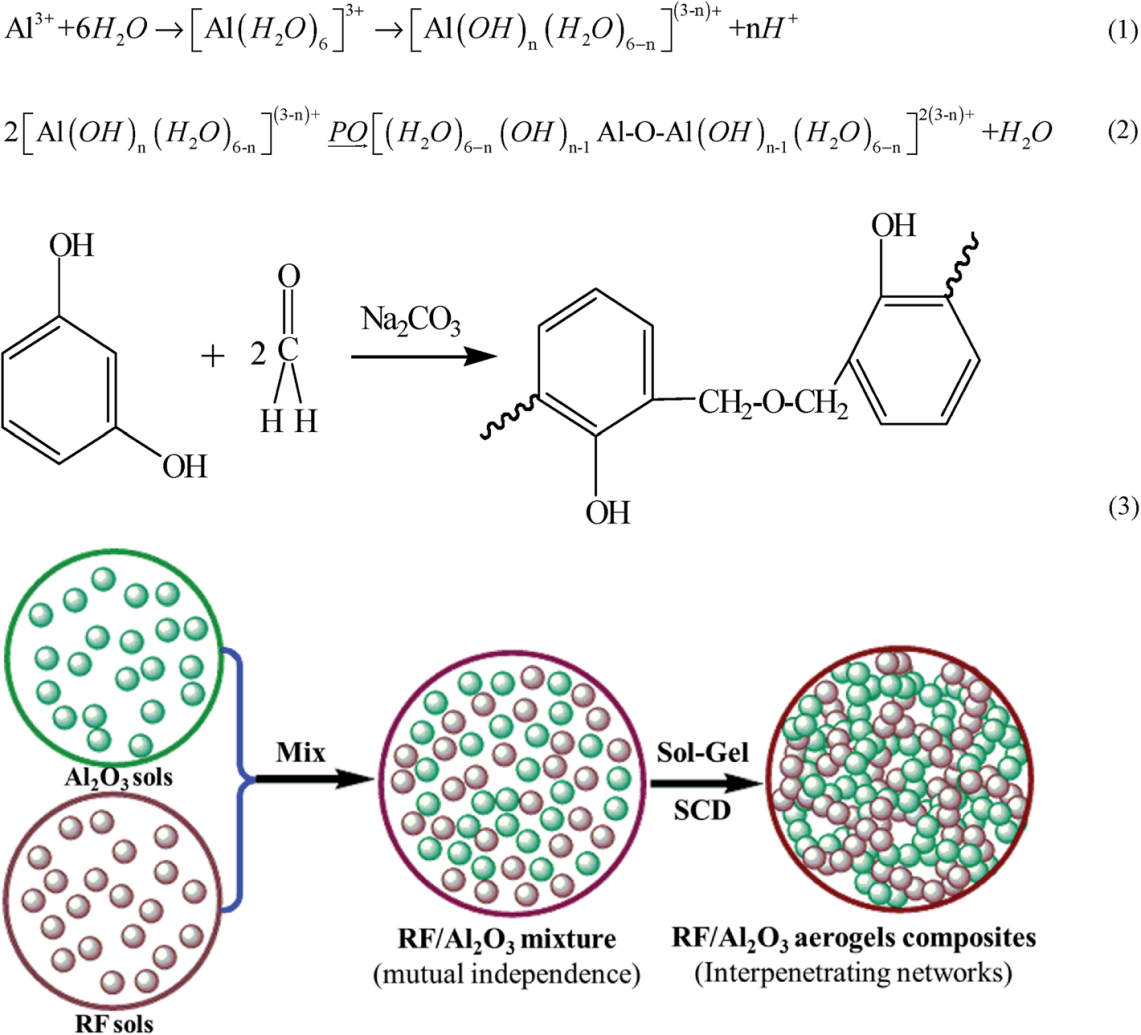
Schematic diagram of the formation mechanism of RF/Al_2_O_3_ aerogels composites.

The reaction mechanisms of the sol–gel process are shown in eqn (1)–(3) and Fig. 1. During the preparation process, AlCl_3_·6H_2_O is utilized as the Al precursor while PO (propylene oxide) is used as the initiator for the hydrolysis and condensation process, leading to the formation of the Al_2_O_3_ gels with three-dimensional network. Meanwhile, resorcinol reacts with formaldehyde to form hydroxymethylated resorcinol using sodium carbonate as catalyst. The hydroxymethyl groups condense with each other to form nanometer-sized RF sols clusters (classified as a phenolic resin), which then crosslink to produce RF gels based on the same chemistry route.^44^ Additionally, both Al_2_O_3_ gelation and RF gelation could occur at room temperature, the epoxide-initiated Al_2_O_3_ gelation in the absence of acid catalysts proceeds faster than the base-catalyzed RF gelation. Finally, the CO_2_ supercritical fluid drying process turns the mutually independent RF/Al_2_O_3_ gels into RF/Al_2_O_3_ aerogel composites, which are in the form of an interpenetrating organic/inorganic networks.

### 3.2 Structural characteristics

**Fig. 2:**
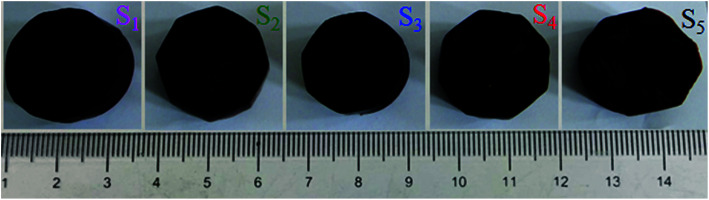
Photographs of RF/Al_2_O_3_ aerogels composites prepared with different RF/Al molar ratios (S_1_: RF/Al = 0.5, S_2_: RF/Al = 0.67, S_3_: RF/Al = 1.0, S_4_: RF/Al = 1.5, S_5_: RF/Al = 2.0).

Fig. 2 shows the photographs of RF/Al_2_O_3_ aerogel composite prepared with different RF/Al molar ratios. All the RF/Al_2_O_3_ aerogel samples are reddish brown in color, and well retain the monolithic morphology after the supercritical fluid CO_2_ drying process. A summary of the textural properties of aerogel samples is represented in Table 1. According to previous reports,^45^ the epoxide-initiated alumina gelation proceeds faster than the base-catalyzed RF gelation at room temperature. With the increase of RF/Al molar ratios, the gelation time of RF/Al_2_O_3_ mixture shows the trend of initial decrease and then increase, which results from the decrease of crystal water in AlCl_3_·6H_2_O and the increase of RF sols. The bulk densities of RF/Al_2_O_3_ aerogel composites are 0.077–0.112 g cm^−3^, while the linear shrinkages are about 1.55–2.76% compared with the original wet gels. The change of bulk densities is attributed to the combined action of RF/Al molar ratios and volume shrinkage. Additionally, all the as-prepared samples with different bulk densities process low thermal conductivities (0.024–0.028 W m^−1^ K^−1^) at 25 °C, which are mainly caused by the unique nanopores and framework structures of the RF/Al_2_O_3_ aerogel composites.

**Table 1:** Summary of the textural properties of RF/Al_2_O_3_ aerogels composites prepared with different RF/Al molar ratios

Sample	Gelation time (min)	Linear shrinkage (%)	Bulk density (g cm^−3^)	Surface areas (m^2^ g^−1^)	Average pore size (nm)	Compressive strength (MPa)	Young's modulus (MPa)	Thermal conductivity (W (m^−1^ K^−1^), 25 °C)
S1	120	1.55	0.094	453.26	48.47	0.74	49.56	0.025
S2	75	2.14	0.103	658.43	39.12	1.29	86.41	0.027
S_3_	50	2.76	0.112	722.75	32.08	1.83	122.57	0.028
S_4_	135	1.97	0.096	643.81	21.57	1.14	75.08	0.026
S_5_	350	1.73	0.077	517.39	17.29	0.56	37.19	0.024

**Fig. 3:**
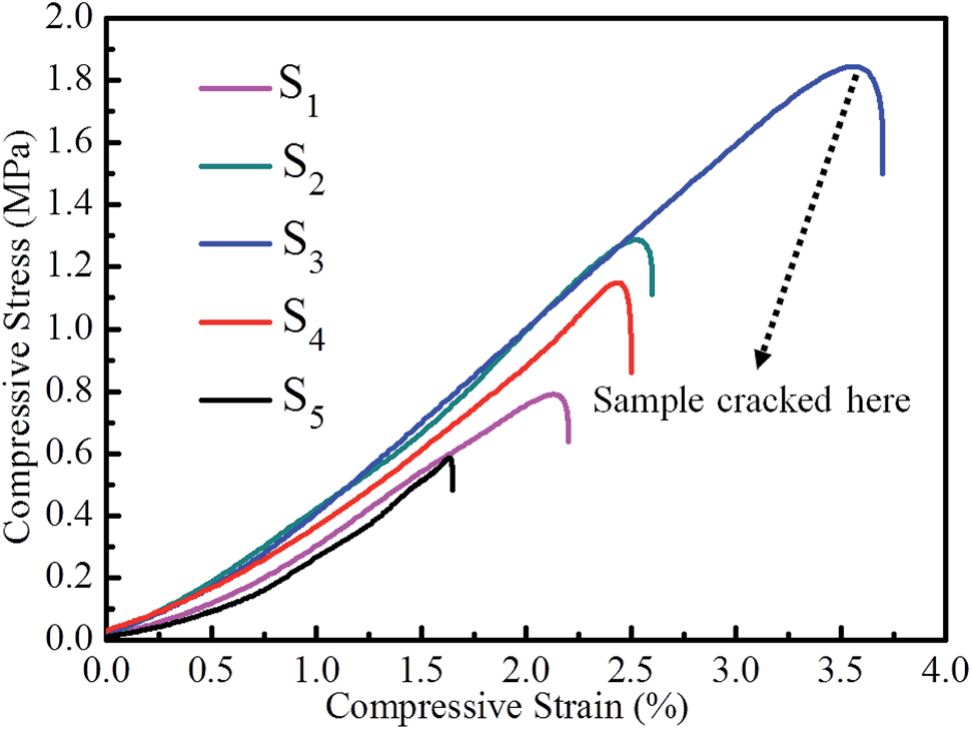
Compressive stress *versus* compressive strain curves of RF/Al_2_O_3_ aerogels composites prepared with different RF/Al molar ratios (S_1_: RF/Al = 0.5, S_2_: RF/Al = 0.67, S_3_: RF/Al = 1.0, S_4_: RF/Al = 1.5, S_5_: RF/Al = 2.0).

Fig. 3 shows the compressive stress *versus* compressive strain curves of RF/Al_2_O_3_ aerogel composites prepared with different RF/Al molar ratios. Table 1 lists the values of compressive strength and Young's modulus of the as-prepared RF/Al_2_O_3_ aerogel composites. The as-synthesized RF/Al_2_O_3_ aerogel composites exhibit an excellent mechanical property, which is one of the highest compressive Young's modulus of pure aerogels without using the structural reinforcement materials (bulk density of about 0.10 g cm^−3^) ever reported. Additionally, it is worth mentioning that the mechanical properties of RF/Al_2_O_3_ aerogel composites with interpenetrating organic/inorganic network structures are closely related to the RF/Al molar ratios. As shown in Fig. 3, the sample with molar ratio RF/Al = 1 shows the best mechanical property, and the values of compressive strength and Young's modulus are 1.83 MPa and 122.57 MPa, respectively, which is mainly caused by more uniform internal framework structure and larger bulk density. It was reported that the aerogels with equal magnitude bulk density about 0.15–0.30 g cm^−3^, such as SiO_2_,^46^ Al_2_O_3_,^47^ TiO_2_,^48^ C_2–50_/SiO_2_,^49^ layer/SiO_2_ (ref. 50) aerogels, exhibited poor mechanical properties (compressive Young's modulus of 3.88 MPa, 11.4 MPa and 3.5 MPa, 23–52 MPa, 8.77 MPa, respectively), which could be due to the low densities as well as the disordered porous network morphology of aerogel materials.

**Fig. 4:**
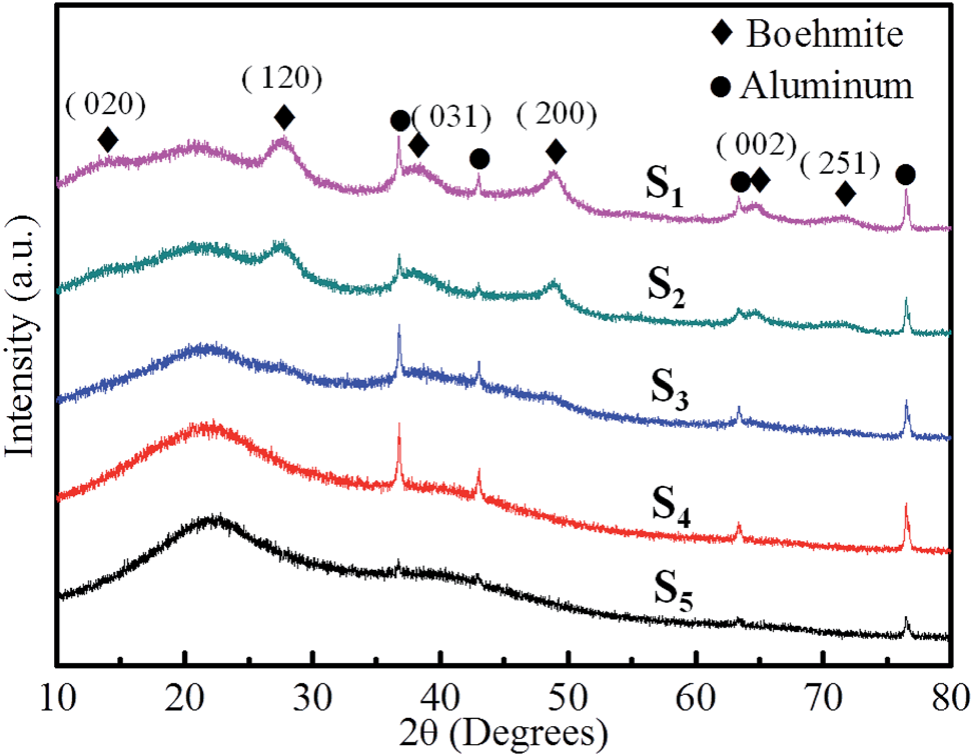
XRD patterns of RF/Al_2_O_3_ aerogels composites prepared with different RF/Al molar ratios (S_1_: RF/Al = 0.5, S_2_: RF/Al = 0.67, S_3_: RF/Al = 1.0, S_4_: RF/Al = 1.5, S_5_: RF/Al = 2.0).

Fig. 4 shows the XRD patterns of RF/Al_2_O_3_ aerogel composites prepared with different RF/Al molar ratios. For all the as-prepared samples, they display relative broad or weak diffraction peaks, indicating the presence of amorphous organic carbon and alumina in RF/Al_2_O_3_ aerogel composites. With the decrease of RF/Al molar ratios, the broad diffraction peak at 22° gradually disappears, meanwhile, the visible characteristic diffraction peaks of boehmite emerge out. The broad diffraction peaks with 2*θ* values of 15°, 28°, 38°, 49°, 65° and 72° correspond to crystal planes of (020), (120), (031), (200), (002), (251) of pseudo-boehmite (AlO(OH), PDF no. 83-2384), respectively. It exists as polycrystalline boehmite instead of the amorphous phase. Furthermore, the ever-present weak peaks at 37°, 43°, 63°, and 76° are due to the poor crystallization γ-Al_2_O_3_ phase in RF/Al_2_O_3_ aerogel composites.

**Fig. 5:**
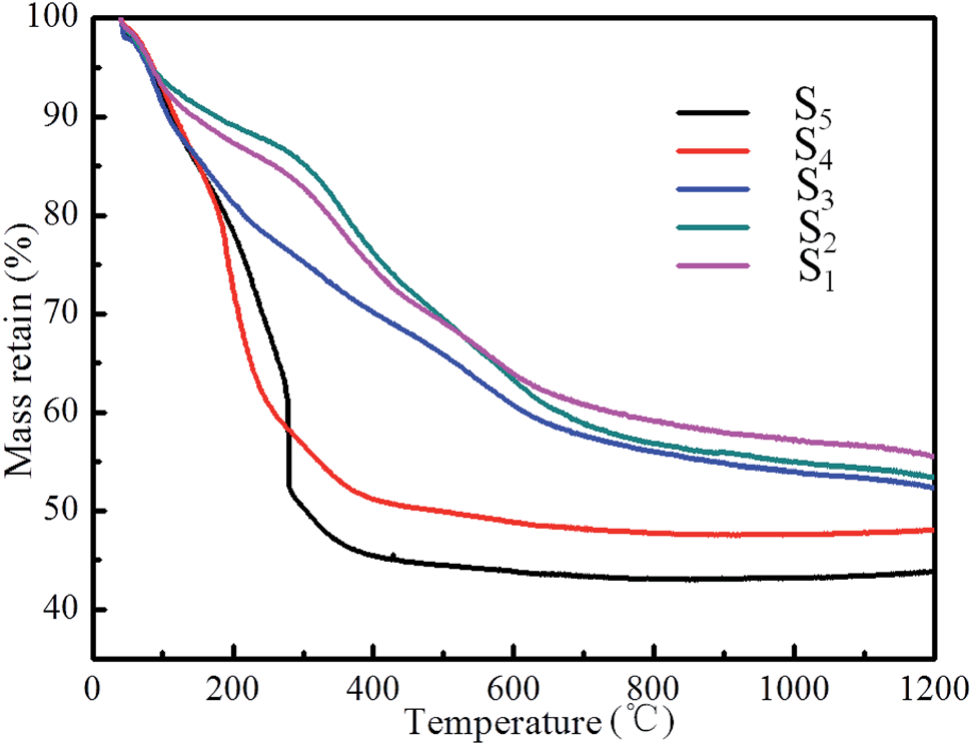
TG of curves of RF/Al_2_O_3_ aerogels composites prepared with different RF/Al molar ratios (S_1_: RF/Al = 0.5, S_2_: RF/Al = 0.67, S_3_: RF/Al = 1.0, S_4_: RF/Al = 1.5, S_5_: RF/Al = 2.0).

Fig. 5 presents the TG curves of the as-dried RF/Al_2_O_3_ aerogel composites prepared with different RF/Al molar ratios heat-treated to 1200 °C in flowing argon. The thermogram profile can be divided into three main regions. The first stage (below 100 °C) is caused by the evolution of physically adsorbed H_2_O, CO_2_ and residual solvent. In general, because of the nano-sized porous structure and high porosity, H_2_O, CO_2_ and solvent adsorbed in the porous structure of the sample can not be removed completely during the supercritical CO_2_ drying process. At the second stage (100–500 °C), an obvious weight loss of all the samples occurs due to the continuous thermal decomposition of the polymer(resorcinol-formaldehyde) in RF/Al_2_O_3_ aerogel composites. For the last stage (above 500 °C), all the TG curves of the RF/Al_2_O_3_ aerogel composites tend to smooth and stabilization. The mass remaining of the samples (S_1_–S_5_) at 1200 °C are 56%, 54%, 53%, 50% and 45% of the original, respectively. According to previous reports,^51^ the TG curves of the samples (S_4_, S_5_) with molar ratios (RF/Al = 1.5, 2.0) similar to the pure RF aerogel under the same conditions, which gives the initial mass loss of adsorbed solvents below 100 °C and only one additional step above 400 °C, yielding at 700 °C a carbon aerogel with a mass loss of ∼50% of the original. In addition, unlike the other mutually interpenetrating resorcinol-formaldehyde/metal oxide (RF/MO_*X*_, M: Fe, Cu) networks,^52^ the TG curves of as-synthesized RF/Al_2_O_3_ aerogel composites suggest that there is no reaction takes place between RF and Al_2_O_3_.

**Fig. 6:**
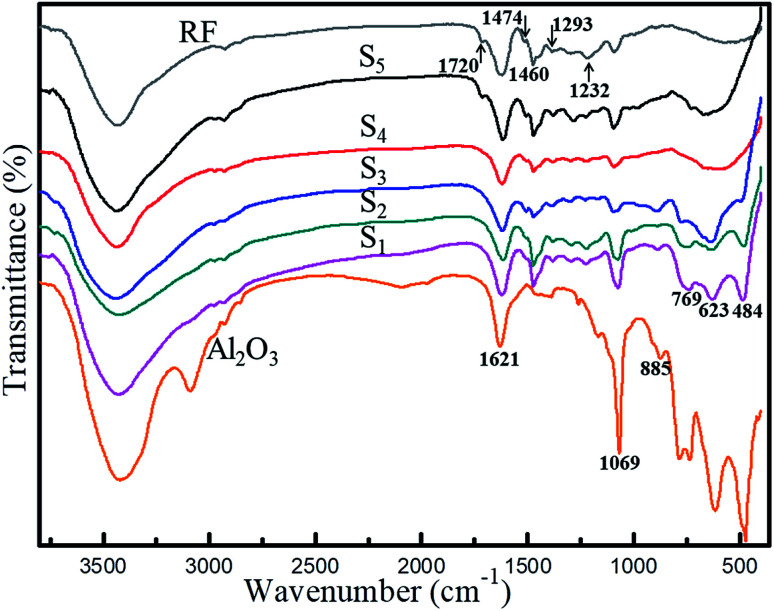
FT-IR spectrum of RF, Al_2_O_3_ aerogel and RF/Al_2_O_3_ aerogels composites (S_1_: RF/Al = 0.5, S_2_: RF/Al = 0.67, S_3_: RF/Al = 1.0, S_4_: RF/Al = 1.5, S_5_: RF/Al = 2.0).

Fig. 6 presents the FT-IR spectrum of polymer (RF), Al_2_O_3_ aerogel and RF/Al_2_O_3_ aerogel composites. As shown in Fig. 6, all the characteristic peaks of the RF/Al_2_O_3_ aerogel composites relatively draw close to each other. Moreover, the characteristic peaks of RF/Al_2_O_3_ aerogel composites are caused by the pure polymer (RF) and Al_2_O_3_ aerogels, and no obvious wavenumber shifts can be observed. The presence of water molecules is evidenced by the bands at 3426 cm^−1^ and 1621 cm^−1^, and there are no significant changes of the intensity with the decrease of RF/Al molar ratios. The two weak bands at 2973 cm^−1^ and 2898 cm^−1^ are related to the C–H stretching vibration of hydrocarbon groups. The unique band only existed in S_5_ at 1720 cm^−1^ is associated with stretching of the C=O bond of carbonyl or carboxyl groups. The bonds at 1460 cm^−1^ and 1474 cm^−1^ are due to the –CH_2_– stretching vibration. The two bonds of 1293 cm^−1^ and 1232 cm^−1^ belong to the stretching vibration of C–O–C hydroxymethyl ether bond. It is well known that the lamellar structure of AlOOH has been previously reported by Yarbrough and Roy.^53^ The band at 1069 cm^−1^ is assigned to the Al–O–H stretching vibration of boehmite. The OH groups within the structure could form zigzag chains between the planes of oxygen ions, which could lead to the OH stretching modes due to their crystallographically inequivalent coupling effect.^54^ The bands at 885 cm^−1^, 769 cm^−1^, 623 cm^−1^ and 484 cm^−1^ are attributed to the Al–O structural vibration of boehmite. It is worth noting that the intensity of the bands corresponding to boehmite weakens gradually with the increase of RF/Al molar ratios, which is caused by the presence of polymer (RF) in its environment. Thus, the above FTIR analysis is consistent with the XRD and TGA results.

**Fig. 7:**
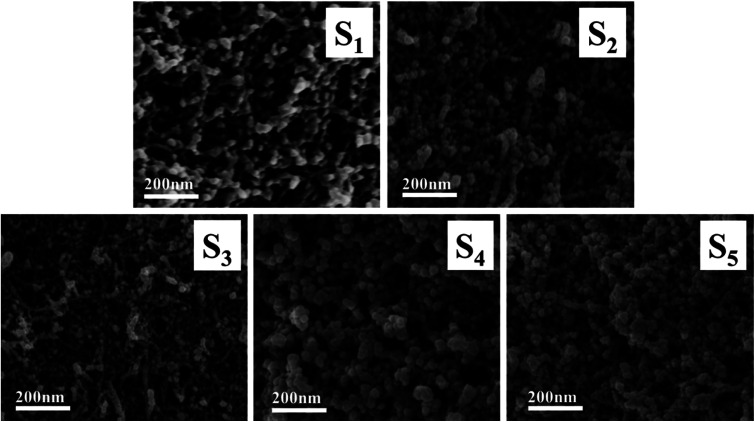
SEM images of RF/Al_2_O_3_ aerogels composites prepared with different RF/Al molar ratios (S_1_: RF/Al = 0.5, S_2_: RF/Al = 0.67, S_3_: RF/Al = 1.0, S_4_: RF/Al = 1.5, S_5_: RF/Al = 2.0).

Fig. 7 shows the microstructures of the RF/Al_2_O_3_ aerogel composites prepared with different RF/Al molar ratios. All the samples exhibited porous structures of a typical colloidal gel, which is consisted of polymer (RF), Al_2_O_3_ nanoparticles and nanopores. It is found that with the increase of RF/Al molar ratios, the morphology gradually changes from pearl-necklace networks to spherical particles. In addition, the as-prepared samples are comprised of interconnected spherical particles with diameters in the 5–15 nm range of polymer (RF) and Al_2_O_3_ aerogel. Furthermore, the nanoparticles of the as-prepared samples progressively adjoin closely to each other and thus possesses the smallest nanopores with diameter at around 10–20 nm for the sample with RF/Al = 2, when compared with the other samples. There are some large pores in the samples with RF/Al = 0.5, 0.67 (S_1_, S_2_), and some agglomeration particles appear in the samples with RF/Al = 1.5, 2.0 (S_4_, S_5_), which is not beneficial for large specific surface areas. In contrast, the sample with RF/Al = 1 (S_3_) exhibits a significant homogeneous pore structures with diameters in the range of approximately 30–40 nm.

**Fig. 8:**
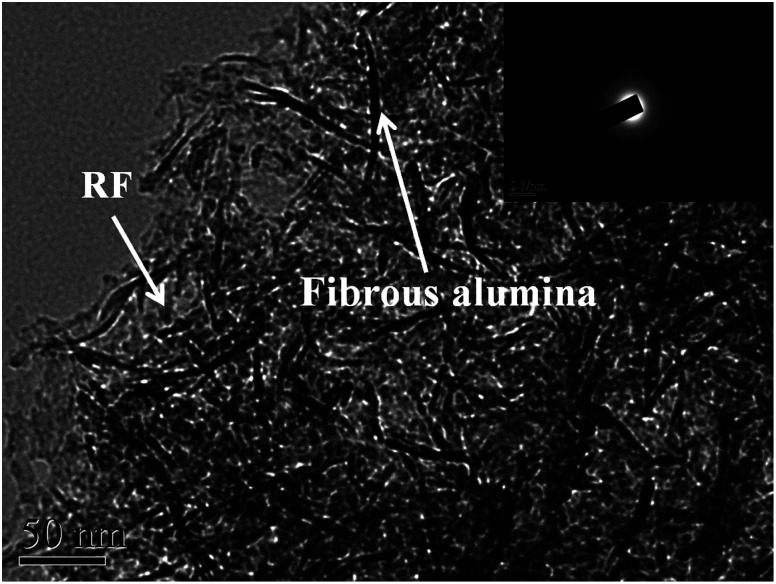
TEM image of RF/Al_2_O_3_ composite and SAED pattern (molar ratio RF/Al = 1).

Transmission electron microscopy (TEM) was employed to further investigate the microstructure of the selected RF/Al_2_O_3_ composite (Fig. 8, molar ratio RF/Al = 1). The TEM image shows that alumina aerogels exhibit randomly interconnected networks made up of nanometer-sized fibrous alumina (dark field), which is similar to leaflets or sheets (2–5 nm wide, varying lengths), and RF aerogels consisted of interconnected amorphous spheroidal particles surrounding the fibrous alumina aerogels. Due to the fibrous alumina existed in RF/Al_2_O_3_ aerogel composites, the mechanical properties of RF/Al_2_O_3_ aerogel composites are further improved. The mechanism could be explained by the similar phenomena occurred in fiber-reinforced system. Intriguingly, unlikely to the other aerogel composites reinforced by thick fibers, the nano-scaled fibrous alumina particles are beneficial to enhancing the mechanical performances of the as-prepared RF/Al_2_O_3_ aerogel composites instead of destroying the internal pore structures.

**Fig. 9:**
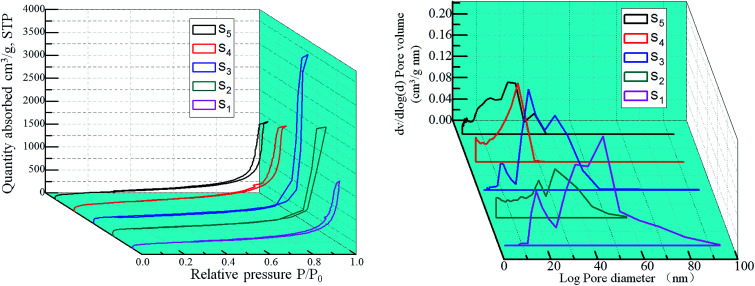
Nitrogen adsorption/desorption isotherms and the pore size distribution curves of RF/Al_2_O_3_ aerogels composites prepared with different RF/Al molar ratios (S_1_: RF/Al = 0.5, S_2_: RF/Al = 0.67, S_3_: RF/Al = 1.0, S_4_: RF/Al = 1.5, S_5_: RF/Al = 2.0).

The adsorption/desorption isotherms and pore size distribution curves of the samples are shown in Fig. 9. They are type IV curves with type H1 hysteresis loop in the IUPAC classification, which is characteristic of a mesoporous structure with cylindrical pores. The desorption cycles of the isotherms show a hysteresis loop for the five samples, which is generally attributed to the capillary condensation that occurs in the mesopores. It is found that with the increase of RF/Al molar ratios, the ranges of the pore size distribution curves are changed from 0–100 nm to 0–30 nm. As shown in Table 1, the values of specific surface areas undergo the trend of first increase and then decrease with the increase of RF/Al molar ratios. Meanwhile, the average pore size of the samples gradually become smaller from 48.47 nm to 17.29 nm. This is because the fact that with the increase of RF/Al molar ratios, the pore structures of the RF/Al_2_O_3_ aerogel composites become more homogenous and some macropore with diameters above 100 nm appear in the composites (S_1_, S_2_, S_3_), which is favorable to increasing the specific surface areas of the composites. By contrast, the sample with RF/Al = 1 shows the preferable framework structure with the highest specific surface areas of 722.75 m^2^ g^−1^ (as shown in Fig. 7 and Table 1). However, some agglomeration particles generate in the composites (Fig. 7) with the continuous increase of RF/Al molar ratio, resulting in the decrease of specific surface area.

## 4. Conclusions

Monolithic RF/Al_2_O_3_ aerogel composites with mutually interpenetrating organic/inorganic network structure were successfully synthesized by a sol–gel method combined with CO_2_ supercritical fluid drying technique. The formation mechanism and the effects of RF/Al molar ratios on structure evolution and physicochemical properties of the RF/Al_2_O_3_ aerogel composites were systematically discussed. The as-prepared samples show uniform mesoporous structures with low bulk density, low thermal conductivity, high specific surface area and excellent mechanical performance, without the use of structural reinforcement materials. Particularly, the sample with molar ratio RF/Al = 1 shows the highest compressive strength and Young's modulus, primarily due to the homogeneous interpenetrating network structure and fibrous alumina reinforcement. Therefore, this novel porous interpenetrating organic/inorganic framework material, consisting of aerogels with outstanding mechanical behavior, offers a broad scope of application in fields requiring the use of aerogel materials.

## Conflicts of interest

The authors declare that there are no conflicts of interest.

## Supplementary Material
